# Effects of arachidonyl-2’-chloroethylamide (ACEA) on the protective action of various antiepileptic drugs in the 6-Hz corneal stimulation model in mice

**DOI:** 10.1371/journal.pone.0183873

**Published:** 2017-08-31

**Authors:** Jarogniew J. Luszczki, Pawel Patrzylas, Miroslaw Zagaja, Marta Andres-Mach, Katarzyna Zaluska, Maria W. Kondrat-Wrobel, Monika Szpringer, Jaroslaw Chmielewski, Magdalena Florek-Luszczki

**Affiliations:** 1 Department of Pathophysiology, Medical University of Lublin, Lublin, Poland; 2 Isobolographic Analysis Laboratory, Institute of Rural Health, Lublin, Poland; 3 Department of Plant Physiology and Biotechnology, The John Paul II Catholic University of Lublin, Lublin, Poland; 4 Faculty of Medicine and Health Sciences, The Jan Kochanowski University in Kielce, Kielce, Poland; 5 Institute of Environmental Protection—National Research Institute, Warszawa, Poland; 6 Department of Gerontology, Institute of Rural Health, Lublin, Poland; University of Modena and Reggio Emilia, ITALY

## Abstract

Accumulating evidence indicates that cannabinoid CB_1_ receptor ligands play a pivotal role in seizures, not only in preclinical studies on animals, but also in clinical settings. This study was aimed at characterizing the influence of arachidonyl-2′-chloroethylamide (ACEA–a selective cannabinoid CB_1_ receptor agonist) co-administered with phenylmethylsulfonyl fluoride (PMSF) on the anticonvulsant potency of various antiepileptic drugs (clobazam, lacosamide, levetiracetam, phenobarbital, tiagabine and valproate) in the 6-Hz corneal stimulation model. Psychomotor seizures in male albino Swiss mice were evoked by a current (32 mA, 6 Hz, 3 s stimulus duration) delivered via corneal electrodes. Potential adverse effects produced by the antiepileptic drugs in combination with ACEA+PMSF were assessed using the chimney test (motor performance), passive avoidance task (remembering and acquisition of learning), and grip-strength test (muscular strength). Brain concentrations of antiepileptic drugs were measured by HPLC to exclude any pharmacokinetic contribution to the observed effect. ACEA (5 mg/kg, i.p.) + PMSF (30 mg/kg, i.p.) significantly potentiated the anticonvulsant potency of levetiracetam (*P*<0.05), but not that of clobazam, lacosamide, phenobarbital, tiagabine or valproate in the 6-Hz corneal stimulation model. Moreover, ACEA+PMSF did not significantly affect total brain concentrations of levetiracetam in mice. No behavioral side effects were observed in animals receiving combinations of the studied antiepileptic drugs with ACEA+PMSF. In conclusion, the combined administration of ACEA+PMSF with levetiracetam is associated with beneficial anticonvulsant pharmacodynamic interaction in the 6-Hz corneal stimulation model. The selective activation of cannabinoid CB_1_ receptor-mediated neurotransmission in the brain may enhance levetiracetam-related suppression of seizures in epilepsy patients, contributing to the efficacious treatment of epilepsy in future.

## Introduction

Despite major advances in the treatment of epilepsy and the availability of a wide range of antiepileptic drugs, there is still more than 30% of patients who still suffer from seizures because of inadequate selection of the antiepileptic drugs to monotherapy [1, 2]. These refractory patients require novel therapeutic and more efficacious strategies involving combined administration of two or even more antiepileptic drugs in order to suppress seizures and improve their quality of living [3, 4].

The flowering tops and leaves as well as resin from the plant *Cannabis sativa* have been used over the centuries as a medicine to help reduce seizures in epileptic patients [5]. Some experimental data confirmed the anticonvulsant activity of natural cannabinoid compounds occurring in *Cannabis sativa* (including, Δ^9^-tetrahydrocannabinol, cannabidiol, cannabidivarin) in various experimental models of epilepsy [6–8]. However, the addictive and psychoactive properties of preparations obtained from this herb totally exclude their direct application in the anticonvulsant therapy [9]. Hence, there is a strong need to look for cannabimimetic substances which would offer antiseizure properties and no harmful side effects.

The latest knowledge from both *in vitro* and *in vivo* studies about the anticonvulsive properties of both synthetic and natural cannabinoid compounds in various experimental models of epilepsy have been summarized recently [10]. Results obtained from many experimental models of epilepsy demonstrated that strong anticonvulsant activity has been evoked by several synthetic cannabimimetic compounds, including WIN 55,212–2 mesylate (WIN—a non-selective cannabinoid CB_1_ and CB_2_ receptor agonist) [11–16], arachidonylcyclopropylamide (ACPA–a specific cannabinoid CB_1_ receptor agonist) [17], and arachidonyl-2’-chloroethylamide (ACEA–a selective cannabinoid CB_1_ receptor agonist) [18–25].

ACEA binds to the cannabinoid CB_1_ receptor with very high affinity, even ~50-fold higher than for endogenous anandamide [26]. ACEA is a structural analog of anandamide, which in physiological conditions can be easily hydrolyzed by intracellular fatty acid amide hydrolase (FAAH) to arachidonic acid and ethanolamine [26, 27]. The enzyme inhibitor, phenylmethylsulfonyl fluoride (PMSF) was proposed to be used as an agent to prolong the time of action of anandamide and other cannabinoid ligands by inhibiting their metabolic degradation [28]. Taking into consideration the above-mentioned facts we used PMSF as an inhibitor of FAAH to protect ACEA from the expected degradation.

Experimental evidence indicates that ACEA combined with PMSF enhanced the anticonvulsant potency of some antiepileptic drugs (i.e. valproate, phenobarbital and pregabalin) in the mouse maximal electroshock-induced tonic seizure model [21–23]. ACEA also potentiated the anticonvulsant effects of ethosuximide, phenobarbital and valproate in the mouse pentylenetetrazole-induced clonic seizure model [20]. Recent studies confirmed also the essential role of another synthetic cannabinoid WIN 55,212–2 mesylate (a non-selective cannabinoid CB_1_ and CB_2_ receptor agonist) in enhancing the anticonvulsant efficacy of various antiepileptic drugs (i.e., gabapentin, levetiracetam, clonazepam, phenobarbital and valproate) in the 6-Hz corneal stimulation model [14, 15]. Considering the above-mentioned facts, it seems interesting and necessary to check the potential anticonvulsant properties of the potent and highly selective cannabinoid CB_1_ receptor agonist—ACEA in combination with various antiepileptic drugs in the 6-Hz corneal stimulation model in mice. Of note, the 6-Hz corneal stimulation model is considered to be an experimental model of partial epilepsy resistant to the treatment with antiepileptic drugs in humans [29, 30].

The objective of this study was to evaluate the effect of ACEA in combination with a constant dose of PMSF on the anticonvulsant potency of several various antiepileptic drugs (clobazam, lacosamide, levetiracetam, phenobarbital, tiagabine and valproate) in the 6-Hz corneal stimulation model in mice. Additionally, we investigated the combination of ACEA with the studied antiepileptic drugs in relation to impairment of long-term memory, motor coordination and skeletal muscular strength in the step-through passive avoidance, chimney and grip-strength tests, respectively. Additionally, total brain antiepileptic drug concentrations were estimated to find out whether the observed anticonvulsant interactions were pharmacodynamic or pharmacokinetic.

## Material and methods

### Animals

Adult male albino Swiss outbred mice (purchased from a licensed breeder Dr. Jacek Kołacz, Warszawa, Poland) were used in all in vivo experiments described herein. After adaptation to laboratory conditions, the mice (8 week-old, weighing 24 ± 3 g) were randomly assigned to experimental groups comprising 8 mice per group. The Second Local Ethics Committee at the University of Life Sciences in Lublin, Poland (Permit Number: 55/2014) has accepted all protocols and procedures involving animals. Experiments complied with the ARRIVE guidelines and were conducted in strict accordance with the EU Directive 2010/63/EU for animal experiments. Total number of mice used in this study was 600 (i.e., 66 groups per 8 mice in the 6-Hz corneal stimulation model [528 mice]; 6 groups per 8 mice in the passive avoidance, chimney and grip-strength tests [48 mice], and 3 groups per 8 mice in the HPLC measurement of brain antiepileptic drug concentrations [24 mice]. Totally, it was 75 groups per 8 mice).

### Drugs

Clobazam (CLB, Sanofi-Aventis Deutschland GmbH, Frankfurt am Main, Germany), lacosamide (LCM, UCB Pharma, Brussels, Belgium), levetiracetam (LEV, UCB Pharma, Braine-l’Alleud, Belgium), phenobarbital (PB, Polfa, Krakow, Poland), tiagabine (TGB, Sanofi Winthrop, Gentilly, France), valproate (magnesium salt–VPA, Sigma-Aldrich, Poznan, Poland), arachidonyl-2′-chloroethylamide (ACEA, Tocris Bioscience, Bristol, UK), and phenylmethylsulfonyl fluoride (PMSF, ICN Biomedicals Inc., Irvine, CA, USA) were used in this study. ACEA and valproate were dissolved in distilled water, whereas the other drugs were suspended in a 1% aqueous solution of Tween 80 (Sigma-Aldrich, Poznan, Poland). All the drugs were administered intraperitoneally (i.p.), in a volume of 5 ml/kg body weight, as follows: ACEA– 10 min, tiagabine– 15 min, PMSF– 20 min, clobazam, lacosamide and valproate– 30 min, levetiracetam and phenobarbital– 60 min before initiation of seizures in the 6-Hz corneal stimulation model, before brain sampling for the measurement of antiepileptic drug concentrations and before behavioral tests assessing long-term memory, motor performance and skeletal muscular strength. The pretreatment times before testing of the antiepileptic drugs reflect the times to the peak of their maximum anticonvulsant effects. Both, the pretreatment times and route of i.p. administration of the drugs were based upon information about their biological activity from the literature and our previous experiments [22, 23, 31–34]. All experiments were conducted by researchers blind to the treatment.

### 6-Hz corneal stimulation model

Psychomotor (limbic) seizures in mice were evoked by current (6 Hz, 0.2 ms rectangular pulse width, 32 mA, 3 s duration) generated by an S48 Square Pulse Stimulator and CCU1 Constant Current Unit (Grass Technologies, West Warwick, RI, USA). After application of ocular anesthetic (0.5% solution of tetracaine hydrochloride) to the mouse corneas, the animals underwent corneal stimulation and were placed separately in Plexiglas cages (25 × 15 × 10 cm) for the observation of the presence or absence of psychomotor seizures, as described previously [14, 15, 35, 36]. Immediately following the 6-Hz corneal stimulation the animals exhibited a “stunned” posture associated with rearing and automatic movements (convulsive and non-convulsive components) that lasted from 60 to 120 s in untreated animals [29, 30, 37, 38]. In this study, seizure activity associated with the 6-Hz corneal stimulation in mice was characterized by both, convulsive and non-convulsive components, including, immobility or stun, jaw and forelimb clonus, twitching of the vibrissae, and an elevated tail or Straub-tail, as mentioned elsewhere [29, 30, 37, 38]. After the seizure activity, the animals resumed their normal exploratory behavior. In our 6-Hz corneal stimulation model, the animals were classified as having seizures when either convulsive or non-convulsive components were observed. Briefly, the mice showing motor seizures or stereotypic movements accompanied with “stunned posture” were considered as non-protected from seizures, as described earlier [14, 15, 35, 36]. In contrast, the mice were considered to be protected if they resumed theirs normal exploratory behavior within 20 s after stimulation. It should be stressed that after the 6-Hz corneal stimulation, the animals were observed for 40 seconds for the absence or presence of either convulsive or non-convulsive signs of psychomotor seizures. When the observation of 8 mice in the respective group was finished, the animals underwent euthanasia by CO_2_ narcosis. To determine median effective doses (ED_50_ values) of antiepileptic drugs, the drugs were administered i.p. at the following doses: clobazam– 0.25, 0.5, 1, 2, 3 mg/kg; lacosamide– 4, 8, 10, 12, 14 mg/kg; levetiracetam– 5, 8, 10, 12.5, 15, 20 mg/kg; phenobarbital– 4, 8, 12, 16 mg/kg; tiagabine– 0.125, 0.25, 0.5, 1 mg/kg; and valproate– 75, 100, 150, 175 mg/kg (S1 Table). Total number of animals used in this procedure was 528.

### Measurement of total brain antiepileptic drug concentrations

Pharmacokinetic estimation of total brain concentrations of antiepileptic drugs was performed only for those combinations of ACEA+PMSF with antiepileptic drugs, whose anticonvulsant effect in the 6-Hz corneal stimulation model was significantly greater than that for control (an antiepileptic drug + vehicle-treated) mice. This was the reason to measure only total brain concentrations of levetiracetam (administered at the dose corresponding to its ED_50_ value in the 6-Hz corneal stimulation model) by HPLC. Briefly, mice after receiving the respective treatment were decapitated at times chosen to coincide with that scheduled for the 6-Hz corneal stimulation model and the whole brains of mice were removed from skulls, weighed, harvested and homogenized using Abbott buffer (1:2 weight/volume) in an Ultra-Turrax T8 homogenizer (IKA Werke, Staufen, Germany). The homogenates were centrifuged at 10,000 *g* for 10 min. and the supernatant samples of 200 μl were added to 200 μl of acetonitrile, vortex-mixed for 1 min., and centrifuged at 10,000 *g* for 15 min. Next, 20 μl of prepared samples was injected into the ODS-2 Hypersil column (5 μm, 150 × 4.6 mm) and analyzed by an automated HPLC system (Dionex, Sunnyvale, CA, USA). The mobile phase was 40 mM triethylammonium phosphate buffer: acetonitrile (400: 600 vol/vol; Fluka, HPLC grade) and its flow rate was established at 1.2 ml/min. Levetiracetam content was detected at 215 nm (wavelength) and the limit of detection of the method was 0.1 μg/ml. Brain concentrations of levetiracetam were expressed in μg/ml of brain supernatants as means ± S.E.M. of 8 separate brain preparations. Total number of mice used in this procedure was 24.

### Grip-strength test

The effects of combinations of ACEA+PMSF with the tested antiepileptic drugs, at doses corresponding to their ED_50_ values from the 6-Hz corneal stimulation model, on skeletal muscular strength in mice were quantified by the grip-strength test, as recommended elsewhere [39, 40]. Because the skeletal muscular strength in mice receiving the combinations of ACEA+PMSF with phenobarbital and valproate, has been estimated in our earlier studies [20, 22, 23], only the combinations of ACEA+PMSF with clobazam, lacosamide, levetiracetam and tiagabine were tested in this study. Briefly, each mouse was lifted by the tail so that its forepaws could grasp a wire stainless steel grid (8 × 8 cm) connected to an electronic dynamometer (BioSeb, Chaville, France). Subsequently, each mouse was gently pulled backward by the tail until the grid was released and maximal force exerted by the mouse before losing the grip was recorded by the grip-strength apparatus. Skeletal muscular strength in mice was expressed in newton (N) as means ± S.E.M. of 8 determinations. Total number of mice used in this procedure was 48.

### Chimney test

The effects of combinations of ACEA+PMSF with the tested antiepileptic drugs, at doses corresponding to their ED_50_ values from the 6-Hz corneal stimulation model, on motor performance in mice were quantified by the chimney test, as recommended elsewhere [41]. Because the motor coordination in mice receiving the combinations of ACEA+PMSF with phenobarbital and valproate, has been estimated in our earlier studies [20, 22, 23], only the combinations of ACEA+PMSF with clobazam, lacosamide, levetiracetam and tiagabine were tested in this study. Briefly, each mouse had to enter into the plastic transparent tube (3 cm inner diameter, 30 cm long) placed horizontally on the table. When the animal reached the opposite end, the plastic tube was vertically positioned like a chimney and the mouse had to climb backwards up in order to escape the tube. The inability of the mouse to climb backwards up the transparent tube within 1 min. indicated the impairment of motor performance. The naïve animals (without any treatment) usually perform this test within 10–20 s. Total number of mice used in this procedure was 48.

### Step-through passive avoidance task

The effects of combinations of ACEA+PMSF with the tested antiepileptic drugs, at doses corresponding to their ED_50_ values from the 6-Hz corneal stimulation model, on long-term memory in mice were quantified by the step-through passive avoidance task, as recommended elsewhere [42, 43]. Because the long-term memory in mice receiving the combinations of ACEA+PMSF with phenobarbital and valproate, has been evaluated in our earlier studies [20, 22, 23], only the combinations of ACEA+PMSF with clobazam, lacosamide, levetiracetam and tiagabine were tested in this study. Briefly, on the first day of experiment after the drugs administration, during the acquisition/conditioning session each mouse was placed in an illuminated (800 lumens) white chamber (10 × 13 × 15 cm) connected to a dark black chamber (25 × 20 × 15 cm) equipped with an electric grid floor. When the mouse after exploring the white chamber crossed to the black chamber, a guillotined door between two chambers was closed and the animal received an electric foot shock (0.6 mA for 6 s), as recommended earlier [43, 44]. After receiving the negative aversive stimulus, the mouse was removed from the dark chamber and returned to the experimental cage equipped with tap water and chow pellet. During the acquisition/conditioning session the mice learn that the moving to the black chamber results in a mild pain. On the following day of experiment (24 h after acquisition session), the animal (without any treatment) during the test session was placed again into the illuminated white chamber and observed up to 180 s in order to evaluate its passive avoidance response (the absence of movement) [45]. The mouse that avoided the black chamber and remained in the bright white chamber for 180 s was considered to have learnt the task. The time that the mouse took to cross to the black chamber was noted. Long-term memory in mice was expressed in seconds (s) as median retention times (with 25th and 75th percentiles) of 8 determinations. Total number of mice used in this experimental procedure was 48.

### Statistical analysis

The ED_50_ values for antiepileptic drugs determined in the 6-Hz corneal stimulation model were calculated by computer log-probit analysis [46] as described in detail earlier [47]. Subsequently, the ED_50_ values (± S.E.M.) were statistically analyzed using one-way analysis of variance (ANOVA) followed by the *post-hoc* Tukey-Kramer test for multiple comparisons. Total brain antiepileptic drug concentrations and the results from the grip-strength test were statistically verified using one-way analysis of variance (ANOVA). The results from the chimney test were compared by use of the Fisher’s exact probability test. The results from the passive avoidance task were statistically analyzed using nonparametric Kruskal-Wallis test. Differences among values were considered statistically significant if *P*<0.05. GraphPad Prism version 7.0 for Windows (GraphPad Software, San Diego, CA, USA) was used as a statistical software.

## Results

### Effect of ACEA and PMSF on the anticonvulsant activity of various antiepileptic drugs in the 6-Hz corneal stimulation model

**Fig 1:**
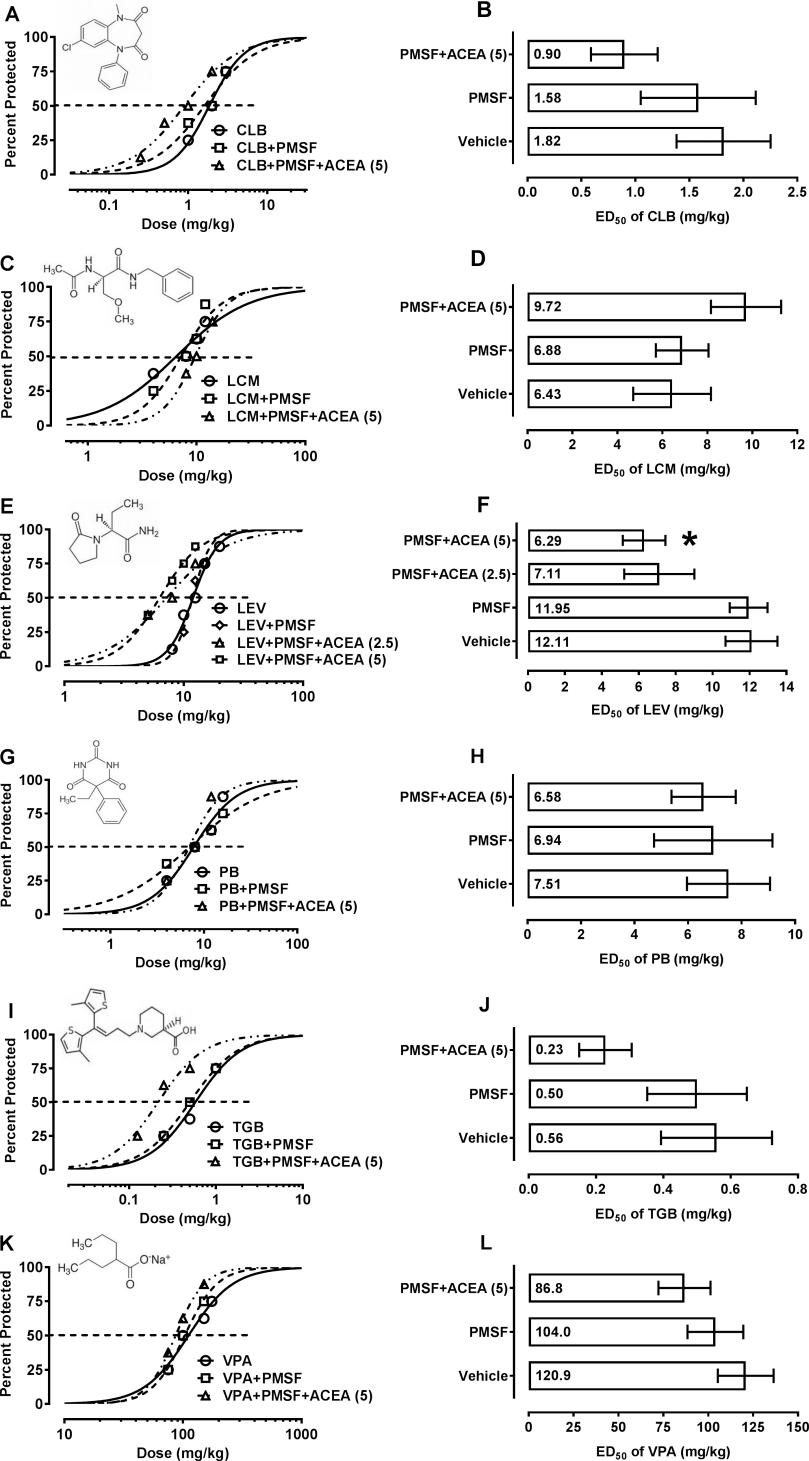
**A-L. Influence of ACEA, PMSF and their combination on the anticonvulsant activity of clobazam (CLB), lacosamide (LCM), levetiracetam (LEV), phenobarbital (PB), tiagabine (TGB) and valproate (VPA) in the 6-Hz corneal stimulation model in mice.** Dose–response functions (sigmoidal curves) for the anticonvulsant activity of various antiepileptic drugs [CLB (A), LCM (C), LEV (E), PB (G), TGB (I) and VPA (K)] alone and in combination with ACEA and PMSF in the 6-Hz corneal stimulation model. Structural formulas of antiepileptic drugs are placed above sigmoidal curves. Each data point corresponds to percent of mice protected (n = 8 mice/data point) from the 6-Hz corneal stimulation model at a given dose (in mg/kg). Points of intersections with the dashed line at 50% reflect approximate ED_50_ values of antiepileptic drugs administered alone and in combination with ACEA and PMSF. Columns represent median effective doses (ED_50_ in mg/kg ± S.E.M.) of antiepileptic drugs [CLB (B), LCM (D), LEV (F), PB (H), TGB (J) and VPA (L)] that protected 50% of the mice from the 6-Hz corneal stimulation model. The log-probit method was used for calculating the ED_50_ values. Data were statistically analyzed with one-way ANOVA and post-hoc Tukey-Kramer test. *P<0.05 *vs*. control (antiepileptic drug+vehicle-treated) animals.

Clobazam, lacosamide, levetiracetam, phenobarbital, tiagabine and valproate when administered alone protected, in a dose-dependent manner, the animals from the 6-Hz corneal stimulation model (Fig 1A–1L, S1 Table). Similarly, the combinations of the studied antiepileptic drugs with PMSF (30 mg/kg) suppressed, in a dose-dependent manner, psychomotor seizures in mice. When ACEA (5 mg/kg) was administered systemically (i.p.) in combination with PMSF (30 mg/kg), they significantly potentiated the anticonvulsant potency of levetiracetam in the 6-Hz corneal stimulation model, by lowering the ED_50_ of levetiracetam by 48% (F(3,92) = 4.812, p = 0.0037; Fig 1F). In contrast, ACEA (2.5 mg/kg) combined with PMSF (30 mg/kg) had no significant impact on the anticonvulsant potency of levetiracetam in the 6-Hz corneal stimulation model. Moreover, ACEA (5 mg/kg) combined with PMSF (30 mg/kg) did not significantly affect the anticonvulsant potency of clobazam, lacosamide, phenobarbital, tiagabine or valproate in the 6-Hz corneal stimulation model (Fig 1A–1L).

### Effect of ACEA and PMSF on total brain antiepileptic drug concentrations

**Fig 2:**
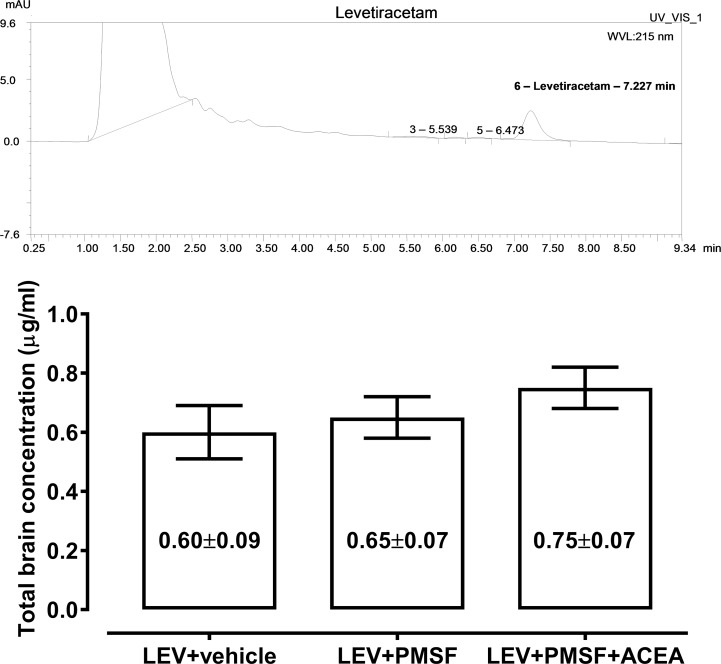
Influence of ACEA and PMSF on total brain concentrations of levetiracetam (LEV) in mice. Columns represent total brain concentrations (means in μg/ml ± S.E.M.) of LEV (n = 8 mice/column). Data were statistically analyzed with one-way ANOVA. Brain tissue samples were taken at times scheduled for the 6-Hz corneal stimulation model.

With HPLC, total brain concentrations of levetiracetam administered alone at a dose of 6.29 mg/kg did not differ significantly from those determined for levetiracetam (6.29 mg/kg) in combination with ACEA (5.0 mg/kg) and PMSF (30 mg/kg) (Fig 2).

### Effects of ACEA and PMSF in combinations with the studied antiepileptic drugs on muscular strength and long-term memory in mice

ACEA (5 mg/kg) and PMSF (30 mg/kg) in combinations with clobazam, lacosamide, levetiracetam and tiagabine (at doses corresponding to their ED_50_ values from the 6-Hz corneal stimulation model) neither altered skeletal muscular strength in mice subjected to the grip strength test, disturbed the acquisition of learning and remembering in mice challenged with the step-through passive avoidance task, nor impaired motor performance in the animals subjected to the chimney test (Table 1).

**Table 1:** Effects of ACEA and PMSF in combinations with clobazam (CLB), lacosamide (LCM), levetiracetam (LEV) and tiagabine (TGB) on long-term memory in the passive avoidance task, muscular strength in the grip-strength test and motor performance in the chimney test in mice.

Treatment (mg/kg)	Retention time (s)	Grip-strength (N)	Impaired motor coordination
**Vehicle + vehicle**	180 (180; 180)	0.842 ± 0.047	0/8
**ACEA (5) + PMSF (30)**	180 (180; 180)	0.829 ± 0.051	0/8
**ACEA (5) + PMSF (30) + CLB (0.90)**	180 (180; 180)	0.834 ± 0.050	0/8
**ACEA (5) + PMSF (30) + LCM (9.72)**	180 (180; 180)	0.837 ± 0.046	0/8
**ACEA (5) + PMSF (30) + LEV (6.29)**	180 (180; 180)	0.850 ± 0.042	0/8
**ACEA (5) + PMSF (30) + TGB (0.23)**	180 (175; 180)	0.839 ± 0.048	0/8

Results are presented as: median retention times (in seconds; with 25^th^ and 75^th^ percentiles in parentheses) from the passive avoidance task, assessing acquisition of learning and remembering in mice; mean strengths (in newtons ± S.E.M.) from the grip-strength test, assessing skeletal muscular strength in mice; and number of mice with impaired motor coordination per total number of the animals in experimental groups challenged with the chimney test, assessing motor performance in mice. Each experimental group consisted of 8 mice. All drugs were administered i.p. at times scheduled from the 6-Hz corneal stimulation model, and at doses corresponding to the ED_50_ value (for more details see Fig 1A–1L).

## Discussion

We found that ACEA (5 mg/kg) co-administered with PMSF (30 mg/kg) significantly potentiated the anticonvulsant action of levetiracetam in mice subjected to the 6-Hz corneal stimulation model. Unfortunately, the anticonvulsant effects of clobazam, lacosamide, phenobarbital, tiagabine and valproate remained almost unchanged after combining these antiepileptic drugs with ACEA and PMSF in the 6-Hz corneal stimulation model. As mentioned earlier, the antiepileptic drugs that protected the mice from seizures in the 6-Hz corneal stimulation model, also efficiently suppress limbic seizures in epilepsy patients, therefore, the 6-Hz corneal stimulation model can be considered an adequate experimental model of psychomotor limbic (drug resistant) seizures in humans [29].

In our 6-Hz corneal stimulation model, the animals were classified as having seizures when either convulsive or non-convulsive components were observed, Briefly, the mice showing motor seizures or stereotypic movements accompanied with “stunned posture” were considered as non-protected from seizures. In contrast, the animals in which normal behavior returned within 20 seconds after seizure initiation were considered as protected from seizures. In our study, there was no need to differentiate between convulsive and non-convulsive psychomotor signs in mice in the 6-Hz corneal stimulation model because, as documented earlier both, convulsive and non-convulsive components correspond to evident pathological changes in the brain electrical activity in experimental mice. More specifically, it has been observed during the EEG recording that both, minimal stereotypies and stunned posture in mice, defined as main non-convulsive signs in the 6-Hz corneal stimulation model, reflected flattering and post-ictal depression of the brain electrical activity in animals [37, 38]. This was the reason to select a recovery to normal behavior within 20 seconds as a criterion that clearly defined a state without any abnormal electric activity in the brains of the mice in the EEG recording. In our study, we did not monitor the animals’ condition after induction of seizures because the mice underwent the 6-Hz corneal stimulation only once, in contrast to the procedure described in details by Giordano et al., [37, 38], who repeatedly stimulated the same mice with inter-stimulation interval of 2–3 days, up to 3–4 times.

It is important to note that in the 6-Hz corneal stimulation model, ACEA at doses of 2.5 and 5 mg/kg combined with PMSF did not significantly affect the threshold for psychomotor seizures (results not shown). However, in our previous experiments we found that ACEA (5 and 7.5 mg/kg) combined with PMSF significantly elevated the threshold for maximal electroconvulsions (tonic seizures) in mice [22], and simultaneously, ACEA (5 and 10 mg/kg) with PMSF had no impact on the threshold for pentylenetetrazole-induced clonic convulsions in mice [20]. Thus, considering the same dose of ACEA (5 mg/kg), it produced different effects depending on the experimental models used, or in other words, some in vivo seizures models may be more sensitive to ACEA doses and activation of cannabinoid CB_1_ receptors by ACEA. Our previous observations, related to the ACEA-mediated effects in the maximal electroshock-induced seizure and pentylenetetrazole-induced seizure tests, have been lately confirmed in the audiogenic seizure model in DBA/2 mice. More specifically, it has been reported that the ED_50_ values of ACEA increased along with the applied seizure models, amounting to 6.87 mg/kg in suppressing tonic phase, 9.38 mg/kg in suppressing clonic phase and 12.68 mg/kg in inhibiting the wild running phase of the audiogenic seizures in DBA/2 mice [48]. This was the main reason to investigate in the present study ACEA in doses of 2.5 and 5 mg/kg that by themselves did not significantly affect the threshold in the 6-Hz corneal stimulation model in mice.

As documented earlier, PMSF was used in our study in a constant dose of 30 mg/kg that also did not change by itself the anticonvulsant threshold in mice and simultaneously it was strong enough to inhibit the enzyme responsible for degradation of anandamide (an endogenous cannabinoid in mammalians) [22, 23]. In the 6-Hz corneal stimulation model, we also found that PMSF (without ACEA) combined with the antiepileptic drugs did not significantly modify the anticonvulsant properties of the studied antiepileptic drugs. This finding confirmed our previous observations showing that PMSF administered alone had no significant influence on the anticonvulsant effects of the antiepileptic drugs in the mouse maximal electroshock-induced and pentylenetetrazole-induced seizure models [20, 22, 23]. Since PMSF was virtually inactive with respect to seizure suppression, the observed potentiation of the anticonvulsant activity of antiepileptic drugs resulted from increased activation of cannabinoid CB_1_ receptor-mediated neurotransmission in the brain of experimental animals. Such the cannabinoid CB_1_ receptor-mediated activation in the animal’s brain resulted probably from both, increased content of anandamide (the endogenous cannabinoid, whose concentration increases after inhibiting the FAAH by PMSF) and the dose of ACEA (the specific cannabinoid CB_1_ receptor agonist). From a theoretical point of view, the inhibitory effect of PMSF on FAAH should contribute to the elevated concentrations of endogenous anandamide in the brain. However, such changes in anandamide concentrations had no significant impact on seizure suppression in mice subjected to the electrically-induced convulsions (maximal electroshock-induced seizure and 6-Hz corneal stimulation models), as it was experimentally observed in our previous studies, reporting no significant effect of PMSF on the threshold for electroconvulsions [22], and threshold in the 6-Hz corneal stimulation model in mice in this study.

On the other hand, the results can be readily compared to those observed earlier in the maximal electroshock-induced seizure test in mice. We have reported that ACEA combined with PMSF potentiated the anticonvulsant action of phenobarbital, valproate and pregabalin in the mouse maximal electroshock-induced seizure model [21–23]. In contrast, ACEA combined with PMSF did not affect the anticonvulsant activity of phenobarbital and valproate in the 6-Hz corneal stimulation model, but enhanced selectively the anticonvulsant properties of levetiracetam. Admittedly, a tendency to potentiation of the antiseizure effects was observed for the combinations of ACEA and PMSF with phenobarbital and valproate in the 6-Hz corneal stimulation model, however, statistical analysis of data did not reach significance. It is worth mentioning that in the maximal electroshock-induced seizure test, valproate and phenobarbital were combined with PMSF and ACEA at doses lower than 5 mg/kg. Since ACEA in the dose of 2.5 mg/kg significantly potentiated the anti-electroshock activity of valproate and phenobarbital in the maximal electroshock-induced seizure test, and simultaneously, ACEA in the dose of 5 mg/kg had no significant impact on the anticonvulsant action of the same antiepileptic drugs in the 6-Hz corneal stimulation model, thus, it can be indirectly ascertained that ACEA-mediated effects depend on seizure models used in preclinical studies (S2 Table).

Furthermore, it was found for the first time that ACEA+PMSF decreased the anticonvulsant action of lacosamide in mice subjected to the 6-Hz corneal stimulation model. In this case, the attenuation of the anticonvulsant properties of lacosamide manifested by a 51% increase in the ED_50_ value of the drug. This unique increase in ED_50_ value for lacosamide did not reach statistical significance with one-way ANOVA. Of note, all the tested antiepileptic drugs except for lacosamide, have had their ED_50_ values slightly reduced as compared to control (antiepileptic drug+vehicle) treated animals.

Additional comparison between the effects observed for ACEA (a selective cannabinoid CB_1_ receptor agonist) and WIN 55,212–2 mesylate (a synthetic non-selective cannabinoid CB_1_ and CB_2_ receptor agonist) can be performed to shed a light on mechanisms responsible for activation of cannabinoid CB_1_ receptors in the 6-Hz corneal stimulation model. As documented earlier, WIN 55,212–2 mesylate potentiated the anticonvulsant activity of clonazepam, gabapentin, levetiracetam, phenobarbital and valproate in experimental animals subjected to the 6-Hz corneal stimulation model [14, 15]. However, in the case of ACEA, the selective and potent cannabinoid CB_1_ receptor agonist enhanced only the anticonvulsant properties of levetiracetam in the 6-Hz corneal stimulation model. In the maximal electroshock-induced seizure test, it was found that WIN 55,212–2 mesylate potentiated the anticonvulsant action of carbamazepine, lamotrigine, phenytoin, phenobarbital, pregabalin, topiramate and valproate [49, 50], while ACEA and PMSF enhanced only the anticonvulsant effects of phenobarbital, pregabalin and valproate in the mouse maximal electroshock-induced seizure model [21–23] (S2 Table). The observed discrepancies between ACEA and WIN 55,212–2 (i.e., selective and non-selective cannabinoid CB_1_ receptor agonists) confirm rather some additional mechanisms of action (unrelated to cannabinoid CB_1_ receptor activation) responsible for WIN 55,212-2-mediated effects in experimental animals.

It this study we have shown that ACEA selectively enhanced the anticonvulsant action of levetiracetam. On the other hand, levetiracetam is considered to be virtually inactive in the maximal electroshock-induced seizure test [51], therefore, its anticonvulsant effects were not investigated in the maximal electroshock-induced seizure model earlier (S2 Table). Pharmacokinetic estimation of total brain levetiracetam concentrations with HPLC in mice receiving the combination of levetiracetam with ACEA and PMSF revealed that neither the combination of ACEA with PMSF nor PMSF alone considerably altered total brain concentrations of levetiracetam in experimental animals, suggesting that the observed beneficial interaction among levetiracetam and ACEA and PMSF was pharmacodynamic in nature.

We also evaluated in this study a potential of antiepileptic drugs combined with ACEA and PMSF to produce acute adverse effects that could be associated with the antiepileptic drug treatment. It is important to note that according to ARRIVE guidelines and the rule of 3Rs (Replacement, Refinement and Reduction of animals in research), we were not allowed to assess adverse effects for the combinations of ACEA+PMSF with phenobarbital and valproate [52]. Previously, we have found that the combinations of ACEA+PMSF with phenobarbital and valproate did not affect long-term memory, grip-strength or motor performance in mice [20, 22, 23]. In our series of behavioral experiments we have found that the combinations of antiepileptic drugs with ACEA and PMSF exerted no significant changes in skeletal muscular strength in animals forcing to grasp the wire mesh in the grip-strength test. Additionally, no significant impairment in acquisition of learning and remembering was observed in mice challenged with the passive avoidance task. No ataxia or motor coordination problems were documented in the animals subjected to the chimney test. However, some additional explanations, concerning the assessment of potential acute adverse effects in animals in a series of behavioral tests in this study, are required. In the step-through passive avoidance task, we determined whether the drug combinations (ACEA+PMSF with antiepileptic drugs) disturbed acquisition and remembering processes in mice. To perform correctly the assessment of long-term memory in animals receiving the cannabinoid receptor CB_1_ agonist (ACEA) and antiepileptic drugs (including, tiagabine), we were obliged to lengthen the time duration of an electric foot shock (aversive stimulation in the acquisition session) because of the antinociceptive properties of antiepileptic drugs [43, 44, 53] and, probably, ACEA. It is highly likely that ACEA, similarly to WIN 55,212–2 mesylate [54], possesses the antinociceptive properties in mice.

In our modification of the passive avoidance task, the time duration of an electric foot shock was prolonged from 2 s (standard variant) to 6 s (modified variant), as recommended earlier [43, 44]. Since tiagabine, vigabatrin, pregabalin, and some cannabinoids evidently exerted the antinociceptive effects in mice, it was reasonably and justified to prolong the time duration of the electrical foot shock in the step-through passive avoidance task [43, 44, 53, 54]. Previously, we have documented that some antiepileptic drugs increase the threshold for the first pain reaction in mice, especially tiagabine, vigabatrin and pregabalin [43, 44, 53], and thus, they may negatively influence the evaluation of long-term memory in mice subjected to the passive avoidance task. To avoid misconduct in the passive avoidance task, we lengthened the time duration of an electric foot shock to ensure that all animals received an adequate negative aversive stimulation when entering the dark compartment that was crucial to properly develop remembering and memory processes in mice, especially, on the first day during the acquisition (training) session in the step-through passive avoidance task [43–45].

Another fact needs explanation. All 3 behavioral tests described hereinabove (i.e., chimney, passive avoidance and grip-strength tests) were conducted on the same animals because of the principles of the 3Rs (Replacement, Reduction and Refinement) in animals’ research. This was the reason to use only 48 mice (6 groups with 8 mice per group) in all 3 behavioral tests. To clearly define the sequence of experiments, each animal (after receiving the respective treatment and at the time to peak of the maximal anticonvulsant effects) was first subjected to the chimney test procedure. After climbing backwards up the plastic tube, each mouse was immediately transferred to the grip-strength apparatus, where skeletal muscular strength of the forelimbs of each mouse was measured. After evaluating muscular strength, the same mouse was inserted into the bright and white chamber of the passive avoidance apparatus, where the animal explored the white illuminated chamber and crossed through the open door into the black chamber. After closing the guillotined door, an aversive electrical stimulus (0.6 mA, 6 s of duration) was generated and the mouse was removed from the dark chamber and returned to the experimental cage. The measurement of retention times was performed 24 hours later in mice inserted again into the white chamber of the passive avoidance apparatus, as described earlier [43, 44]. It should be stressed that the testing of the motor coordination and skeletal muscular strength was performed on the first day of experiments while the evaluation of the long-term memory (the test session) was performed on the next day (24 hours after training session). Of note, to assess acute adverse effects in the chimney and grip-strength tests as well as to train one animal in the acquisition of the step-through passive avoidance test, the experienced experimenter needed approx. 2–3 minutes for each animal. Since the time to peak of maximum effect of ACEA was 10 minutes, we were obliged to split each experimental group into 2 subgroups for 4 mice and all 8 mice were marked with non-toxic markers and the gap period between administration of drugs in particular subgroups was established at 10 minutes.

Thus, considering the above-mentioned results, it can be assumed that the combinations did not alter the animals’ behavior and thus, they can be considered to be safe and worthy of recommendation to clinical conditions. On the other hand, it is important to note that all behavioral tests applied in this study were sensitive enough to detect any significant changes in animals. For instance, we have reported earlier a significant impairment in memory processes in mice receiving vigabatrin and tiagabine and subjected to the passive avoidance task [43, 44]. We have also reported an increased skeletal muscular strength in mice injecting with sildenafil (a selective phosphodiesterase 5 (PDE5) inhibitor) either alone or in combination with antiepileptic drugs [55]. Thus, the above-discussed facts confirm the usefulness of these behavioral tests in investigating potential side effects occurring when combining antiepileptic drugs with some tested compounds that affect central nervous system.

## Conclusions

If the results from this preclinical study could be translated to clinical conditions, some patients with limbic seizures would benefit from the combinations of levetiracetam with ACEA+PMSF. Potentiation of the anticonvulsant effects of levetiracetam by ACEA+PMSF, lack of any acute adverse effects in animals and no pharmacokinetic interactions among the tested drugs, make the combination of levetiracetam with ACEA+PMSF of crucial importance for further clinical practice.

## Supporting information

S1 Table Anticonvulsant activity of clobazam (CLB), lacosamide (LCM), levetiracetam (LEV), phenobarbital (PB), tiagabine (TGB), valproate (VPA) alone and in combination with PMSF and ACEA in the 6-Hz corneal stimulation model. First column represents doses of particular antiepileptic drugs used in the 6-Hz corneal stimulation model. Results are presented as numbers of animals protected from seizures per total numbers of animals in the experimental groups.
(DOC)

S2 Table Influence of ACEA on the anticonvulsant action of the studied antiepileptic drugs in various animal seizure models. Doses of ACEA that significantly potentiated the anticonvulsant activity of the studied antiepileptic drugs are presented in parentheses. MES–maximal electroshock-induced seizure test (tonic-clonic seizures), PTZ–pentylenetetrazole-induced seizure test (myoclonic seizures), 6 Hz–the 6-Hz corneal stimulation model (limbic seizures), ↑ –increase in the anticonvulsant activity of the studied antiepileptic drug. 0 –no significant effect despite the administration of ACEA at a maximally tested dose. N.T.–not tested.
^a^–results from [21],
^b^–results from [23],
^c^–results from [22],
^d^–results from [20],
^e^–results from this study.
(DOC)

## Abbreviations

ACEA: arachidonyl-2′-chloroethylamide
PMSF: phenylmethylsulfonyl fluoride
