# Supplementary material for: Effects of arachidonyl-2’-chloroethylamide (ACEA) on the protective action of various antiepileptic drugs in the 6-Hz corneal stimulation model in mice
Source: PLoS One. 2017 Aug 31;12(8):e0183873. doi: 10.1371/journal.pone.0183873 (PMC5578658; doi:10.1371/journal.pone.0183873)
Supplement: S1 Table — First column represents doses of particular antiepileptic drugs used in the 6-Hz corneal stimulation model. Results are presented as numbers of animals protected from seizures per total numbers of animals in the experimental groups. (DOC) [file pone.0183873.s001.doc]

**S1 Table. Anticonvulsant activity of clobazam (CLB), lacosamide (LCM), levetiracetam (LEV), phenobarbital (PB), tiagabine (TGB), valproate (VPA) alone and in combination with PMSF and ACEA in the 6-Hz corneal stimulation model.**

| **Dose of CLB (mg/kg)** | **CLB+vehicle** | **CLB+PMSF** | **CLB+PMSF+ACEA (5)** |
| --- | --- | --- | --- |
| **0.25** |  |  | 1/8 |
| **0.5** |  |  | 3/8 |
| **1** | 2/8 | 3/8 | 4/8 |
| **2** | 4/8 | 4/8 | 6/8 |
| **3** | 6/8 | 6/8 |  |

| **Dose of LCM (mg/kg)** | **LCM+vehicle** | **LCM+PMSF** | **LCM+PMSF+ACEA (5)** |
| --- | --- | --- | --- |
| **4** | 3/8 | 2/8 |  |
| **8** | 4/8 | 4/8 | 3/8 |
| **10** | 5/8 | 5/8 | 4/8 |
| **12** | 6/8 | 7/8 |  |
| **14** |  |  | 6/8 |

| **Dose of LEV (mg/kg)** | **LEV+vehicle** | **LEV+PMSF** | **LEV+PMSF+ACEA (2.5)** | **LEV+PMSF+ACEA (5)** |
| --- | --- | --- | --- | --- |
| **5** |  |  | 3/8 | 3/8 |
| **8** | 1/8 |  | 4/8 | 5/8 |
| **10** | 3/8 | 2/8 |  | 6/8 |
| **12.5** | 4/8 | 5/8 | 6/8 | 7/8 |
| **15** | 6/8 | 6/8 |  |  |
| **20** | 7/8 |  |  |  |

| **Dose of PB (mg/kg)** | **PB+vehicle** | **PB+PMSF** | **PB+PMSF+ACEA (5)** |
| --- | --- | --- | --- |
| **4** | 2/8 | 3/8 | 2/8 |
| **8** | 4/8 | 4/8 | 4/8 |
| **12** | 5/8 | 5/8 | 7/8 |
| **16** | 7/8 | 6/8 |  |

| **Dose of TGB (mg/kg)** | **TGB+vehicle** | **TGB+PMSF** | **TGB+PMSF+ACEA (5)** |
| --- | --- | --- | --- |
| **0.125** |  |  | 2/8 |
| **0.25** | 2/8 | 2/8 | 5/8 |
| **0.5** | 3/8 | 4/8 | 6/8 |
| **1** | 6/8 | 6/8 |  |

| **Dose of VPA (mg/kg)** | **VPA+vehicle** | **VPA+PMSF** | **VPA+PMSF+ACEA (5)** |
| --- | --- | --- | --- |
| **75** | 2/8 | 2/8 | 3/8 |
| **100** | 4/8 | 4/8 | 5/8 |
| **150** | 5/8 | 6/8 | 7/8 |
| **175** | 6/8 |  |  |

First column represents doses of particular antiepileptic drugs used in the 6-Hz corneal stimulation model. Results are presented as numbers of animals protected from seizures per total numbers of animals in the experimental groups.
