# Supplementary material for: Effects of arachidonyl-2’-chloroethylamide (ACEA) on the protective action of various antiepileptic drugs in the 6-Hz corneal stimulation model in mice
Source: PLoS One. 2017 Aug 31;12(8):e0183873. doi: 10.1371/journal.pone.0183873 (PMC5578658; doi:10.1371/journal.pone.0183873)
Supplement: S2 Table — Doses of ACEA that significantly potentiated the anticonvulsant activity of the studied antiepileptic drugs are presented in parentheses. MES–maximal electroshock-induced seizure test (tonic-clonic seizures), PTZ–pentylenetetrazole-induced seizure test (myoclonic seizures), 6 Hz–the 6-Hz corneal stimulation model (limbic seizures), ↑ –increase in the anticonvulsant activity of the studied antiepileptic drug. 0 –no significant effect despite the administration of ACEA at a maximally tested dose. N.T.–not tested. a–results from [21], b–results from [23], c–results from [22], d–results from [20], e–results from this study. (DOC) [file pone.0183873.s002.doc]

1. **S2 Table. Influence of ACEA on the anticonvulsant action of the studied antiepileptic drugs in various animal seizure models.**

| 1. **Antiepileptic drug** | 1. **Seizure model** | | |
| --- | --- | --- | --- |
|  | 1. **MES** | 1. **PTZ** | 1. **6 Hz** |
| 1. **Clobazam** | 1. 0 (2.5 mg/kg) ^a^ | 1. N.T. | 1. 0 (5 mg/kg) ^e^ |
| 1. **Lacosamide** | 1. 0 (2.5 mg/kg) ^a^ | 1. N.T. | 1. 0 (5 mg/kg) ^e^ |
| 1. **Levetiracetam** | 1. N.T. | 1. N.T. | 1. ↑ (5 mg/kg) ^e^ |
| 1. **Phenobarbital** | 1. ↑ (2.5 mg/kg) ^b^ | 1. ↑ (10 mg/kg) ^d^ | 1. 0 (5 mg/kg) ^e^ |
| 1. **Tiagabine** | 1. N.T. | 1. N.T. | 1. 0 (5 mg/kg) ^e^ |
| 1. **Valproate** | 1. ↑ (2.5 mg/kg) ^c^ | 1. ↑ (10 mg/kg) ^d^ | 1. 0 (5 mg/kg) ^e^ |

1. Doses of ACEA that significantly potentiated the anticonvulsant activity of the studied antiepileptic drugs are presented in parentheses. MES – maximal electroshock-induced seizure test (tonic-clonic seizures), PTZ – pentylenetetrazole-induced seizure test (myoclonic seizures), 6 Hz – the 6-Hz corneal stimulation model (limbic seizures), ↑ – increase in the anticonvulsant activity of the studied antiepileptic drug. 0 – no significant effect despite the administration of ACEA at a maximally tested dose. N.T. – not tested.
2. ^a^ – results from [21],
3. ^b^ – results from [23],
4. ^c^ – results from [22],
5. ^d^ – results from [20],
6. ^e^ – results from this study
